# Gayetty Outdone

**Published:** 1867-07

**Authors:** 


***Gayetty Outdone.***

A certain Dr. D—l avers in the "Healer's Advocate:"

"Our healing virtue assimilates, more or less, with material matter that is brought in contact with us. Hence frequently remarkable cures have been effected in one family where but one patient had been treated. *Invalids have sent us garments*, which, handled by us, would become imbued with our healing virtue, and have been cured by them. We have often furnished invalids with imbued paper, and have helped many thereby."

This creature, instead of being flogged at the cart's tail out of town, is to-day laying on his hands and applying his "imbued paper" in many fashionable quarters of Chicago. Several wondering blockheads have asked us if we didn't think the d—l was in it? To which we have modestly replied that it was only another evidence of the tendency of the human mind to proceed to extremities—to get at the very *bottom* of things. Can human credulity go further?

